# Supplementary material for: Reduction of misdiagnosis in urinary tract infections during pregnancy: The role of adjusted urine flow cytometry parameters
Source: PLoS One. 2024 Sep 23;19(9):e0308253. doi: 10.1371/journal.pone.0308253 (PMC11419365; doi:10.1371/journal.pone.0308253)
Supplement: S1 Table — (DOCX) [file pone.0308253.s001.docx]

**S1 Table. Correlations between Age, gestational age, red blood cells, white blood cells, yeast, bacteria, epithelium**

| 镜检 |  |  |  |  |  |  |  |  | uf5000 |  |  |  |  |  |  |  |  |  |
| --- | --- | --- | --- | --- | --- | --- | --- | --- | --- | --- | --- | --- | --- | --- | --- | --- | --- | --- |
| **Correlations** | | | | | | | | |  | **Correlations** | | | | | | | | |
|  |  | Age | gestational age | red blood cells | white blood cells | yeast | bacteria | epithelium |  |  |  | Age | gestational age | red blood cells | white blood cells | yeast | bacteria | epithelium |
| Spearman's rho | Age | 1.000 | -0.026 | 0.014 | -.096^*^ | 0.001 | -0.037 | -0.043 |  | Spearman's rho | Age | 1.000 | -0.026 | 0.030 | -.125^**^ | -0.012 | -0.043 | -0.081 |
|  |  |  | 0.553 | 0.749 | 0.026 | 0.982 | 0.384 | 0.319 |  |  |  |  | 0.553 | 0.478 | 0.003 | 0.774 | 0.319 | 0.059 |
|  | gestational age | -0.026 | 1.000 | .104^*^ | .187^**^ | -0.037 | -0.065 | -.132^**^ |  |  | gestational age | -0.026 | 1.000 | 0.007 | .124^**^ | -0.077 | -.149^**^ | -.123^**^ |
|  |  | 0.553 |  | 0.016 | 0.000 | 0.384 | 0.128 | 0.002 |  |  |  | 0.553 |  | 0.869 | 0.004 | 0.074 | 0.001 | 0.004 |
|  | red blood cells | 0.014 | .104^*^ | 1.000 | .463^**^ | 0.029 | .134^**^ | .131^**^ |  |  | red blood cells | 0.030 | 0.007 | 1.000 | .393^**^ | .405^**^ | .277^**^ | .240^**^ |
|  |  | 541 | 539 | 541 | 540 | 541 | 541 | 541 |  |  |  | 545 | 543 | 545 | 545 | 545 | 545 | 545 |
|  | white blood cells | -.096^*^ | .187^**^ | .463^**^ | 1.000 | .126^**^ | .400^**^ | .451^**^ |  |  | white blood cells | -.125^**^ | .124^**^ | .393^**^ | 1.000 | .564^**^ | .636^**^ | .597^**^ |
|  |  | 0.026 | 0.000 | 0.000 |  | 0.003 | 0.000 | 0.000 |  |  |  | 0.003 | 0.004 | 0.000 |  | 0.000 | 0.000 | 0.000 |
|  | yeast | 0.001 | -0.037 | 0.029 | .126^**^ | 1.000 | .094^*^ | .196^**^ |  |  | yeast | -0.012 | -0.077 | .405^**^ | .564^**^ | 1.000 | .653^**^ | .557^**^ |
|  |  | 0.982 | 0.384 | 0.501 | 0.003 |  | 0.029 | 0.000 |  |  |  | 0.774 | 0.074 | 0.000 | 0.000 |  | 0.000 | 0.000 |
|  | bacteria | -0.037 | -0.065 | .134^**^ | .400^**^ | .094^*^ | 1.000 | .514^**^ |  |  | bacteria | -0.043 | -.149^**^ | .277^**^ | .636^**^ | .653^**^ | 1.000 | .658^**^ |
|  |  | 0.384 | 0.128 | 0.002 | 0.000 | 0.029 |  | 0.000 |  |  |  | 0.319 | 0.001 | 0.000 | 0.000 | 0.000 |  | 0.000 |
|  | epithelium | -0.043 | -.132^**^ | .131^**^ | .451^**^ | .196^**^ | .514^**^ | 1.000 |  |  | epithelium | -0.081 | -.123^**^ | .240^**^ | .597^**^ | .557^**^ | .658^**^ | 1.000 |
|  |  | 0.319 | 0.002 | 0.002 | 0.000 | 0.000 | 0.000 |  |  |  |  | 0.059 | 0.004 | 0.000 | 0.000 | 0.000 | 0.000 |  |
| *. Correlation is significant at the 0.05 level (2-tailed). |  |  |  |  |  |  |  |  |  | **. Correlation is significant at the 0.01 level (2-tailed). |  |  |  |  |  |  |  |  |
| **. Correlation is significant at the 0.01 level (2-tailed). |  |  |  |  |  |  |  |  |  |  |  |  |  |  |  |  |  |  |
